# Balancing Act: Evaluating the Impact of Direct Oral Anticoagulants on Gastrointestinal Complications in Atrial Fibrillation Patients

**DOI:** 10.7759/cureus.83298

**Published:** 2025-05-01

**Authors:** Rakahn Haddadin, Steven Molina, Srusty Patel, George Trad, John Ryan, Pinak Shah

**Affiliations:** 1 Medicine, MountainView Hospital, Las Vegas, USA; 2 Internal Medicine, University of California Riverside School of Medicine, Riverside, USA; 3 Neurology, St. George's University School of Medicine, St. George's, GRD; 4 Internal Medicine, MountainView Hospital, Las Vegas, USA; 5 Gastroenterology, Southern Hills Hospital and Medical Center, Las Vegas, USA

**Keywords:** acute gi bleed, atrial fib, atrial fibrillation management, oral anticoagulation, upper gastrointestinal (ugi) bleeding

## Abstract

Atrial fibrillation (AF) is commonly treated with anticoagulant therapy, which is effective in reducing stroke risk. However, it can increase the likelihood of gastrointestinal (GI) complications. The literature on GI bleeding related to direct oral anticoagulants (DOACs) has expanded significantly since their approval, revealing both benefits and risks. Data from 66 healthcare organizations across the United States in the TriNetX database were studied. Adult patients with a history of AF and a subsequent history of GI bleeding were included. The study population was divided into pre-2010 and post-2010 cohorts. Our results showed there were statistically significant differences between patients in the post-2010 group and those in the pre-2010 group in their need for blood transfusion. There was also a statistically significant difference in the number of patients who needed an upper endoscopy. Patients who had an International Classification of Diseases (ICD)-10 code of any type of shock showed statistical significance with more patients having shock as a diagnosis after 2010 versus before. The results of this study reveal significant clinical implications following the introduction of DOACs in managing AF. The increase in blood transfusions likely reflects heightened bleeding risks associated with DOACs, particularly in patients with pre-existing GI issues. Additionally, the rise in upper endoscopies suggests clinicians have become more proactive in investigating GI complications, driven by increased awareness of anticoagulant risks.

## Introduction

Atrial fibrillation (AF) is the most common cardiac arrhythmia, affecting an estimated 33 million people worldwide, with its prevalence continuing to rise, particularly in aging populations [1]. In the United States alone, AF affects about 2.7-6.1 million individuals, and this number is projected to increase to 12 million by 2030 due to the growing elderly population [2,3]. Given the significant risk of stroke associated with AF, an estimated fivefold increase compared to the general population, anticoagulation therapy is considered to be the mainstay of management to prevent thromboembolic events [4]. Direct oral anticoagulants (DOACs), such as rivaroxaban, apixaban, edoxaban, and dabigatran, have become first-line treatments for stroke prevention in patients with non-valvular AF due to several advantages over traditional therapy with warfarin. DOACs have been shown to have a more predictable pharmacokinetic profile, require less frequent monitoring of international normalized ratio (INR) values, and have fewer food and drug interactions than warfarin [5,6]. Consequently, DOACs have been increasingly adopted as the preferred anticoagulation strategy for many patients with AF, with guidelines from major cardiovascular societies recommending them over warfarin in most cases [5,7]. Clinical trials such as the RE-LY, ROCKET AF, ARISTOTLE, and ENGAGE AF-TIMI 48 have demonstrated that DOACs are as effective as warfarin in reducing the risk of stroke or systemic embolism and are associated with a lower risk of intracranial hemorrhage [8-10].

While DOACs have become a first-line treatment for stroke prevention in AF due to their favorable safety profile compared to warfarin, the risk of gastrointestinal (GI) bleeding remains a significant concern. The primary mechanism by which DOACs increase the risk of GI bleeding is by inhibiting specific clotting factors. For example, rivaroxaban and apixaban inhibit factor Xa, and dabigatran directly inhibits thrombin (factor IIa), preventing the formation of fibrin clots. Post-approval surveillance and real-world studies have indicated an increased incidence of GI bleeding in patients treated with DOACs, particularly among older adults and those with pre-existing GI conditions [11,12]. For example, a population-based cohort study comparing dabigatran, rivaroxaban, and warfarin found that both dabigatran and rivaroxaban were associated with a similar risk of GI bleeding when compared to warfarin, especially in patients over 75 years of age [11]. Given these adverse effects of DOACs, there is a critical need to further evaluate how the introduction of these agents has impacted overall clinical outcomes in patients with AF.

This study aims to investigate the rates of GI bleeding in patients with AF before and after the implementation of DOACs. DOACs were introduced into clinical practice following their Food and Drug Administration (FDA) approval, with dabigatran first approved in 2010, followed by rivaroxaban in 2011, apixaban in 2012, and edoxaban in 2015 [13]. Given this time frame, the pre- and post-DOAC introduction period comparison would offer crucial insight into whether the adoption of these anticoagulants has resulted in a significant increase in GI bleeding events, necessitating more frequent surveillance with endoscopic interventions or hospitalizations. The primary objective of this study was to evaluate the safety profile of abelacimab in terms of bleeding events in patients with AF, compared to rivaroxaban. Furthermore, examining the prevalence of severe outcomes associated with DOAC use, like hemorrhagic shock, can help clinicians better understand the risk-benefit profile of DOACs and guide safer prescribing practices.

## Materials and methods

Data source

We conducted a comprehensive analysis using data from the TriNetX database. The TriNetX database is a global federated health research network providing access to electronic medical records across large healthcare organizations (HCOs). This data was pulled from 66 HCOs.

Ethical compliance with human studies

This retrospective study is exempt from informed consent. The data reviewed is a secondary analysis of existing data, does not involve intervention or interaction with human subjects, and is de-identified per the de-identification standard defined in Section §164.514(a) of the Health Insurance Portability and Accountability Act of 1996 (HIPAA) Privacy Rule. The process by which the data is de-identified is attested to through a formal determination by a qualified expert as defined in Section §164.514(b)(1) of the HIPAA Privacy Rule. This formal determination by a qualified expert was refreshed in December 2020.

Study population

TriNetX was queried using the International Classification of Diseases (ICD)-9 and ICD-10-CM codes. Patients who were hospitalized with a diagnosis of AF and/or atrial flutter from 1899 to January 1, 2010, were in cohort 1, while patients hospitalized with a diagnosis of AF and flutter after January 2, 2010, were in cohort 2. Any instance of GI bleeding that occurred at least seven days after the first instance of AF was used. Patients were excluded from the study if they had any neoplasms, cirrhotic liver disease, or bleeding diathesis, and the following ICD-10 codes were used for definition: ICD-10 C73-C75, C7A, C76-C80, C81-96, C60-C63, C69-C72, C64-68, C51-58, C50, C45-C49, C43-44, C40-41, C15-C26, K74, C30-39, D65-D69, and C00-C14. A total of 13,747 patients were found in the pre-2010 cohort, while a total of 107,330 patients were found in the post-2010 cohort.

Study outcomes and variables

There were a total of six primary outcomes. We analyzed the two cohorts and looked at the need for transfusion, endoscopy, colonoscopy, death, shock, and acute kidney injury (AKI). ICD-10 codes, procedure codes, and Current Procedural Terminology (CPT) codes were used to identify the outcomes.

Statistical analysis

To minimize bias and ensure comparability between the groups, we employed propensity score matching. This statistical technique was used to create matched pairs of patients from the pre-2010 and post-2010 cohorts based on a range of covariates, including age, sex, race, and baseline renal function. The goal was to create two groups that were similar with respect to these baseline characteristics, thereby isolating the effect of DOACs on GI bleeding and related complications.

Following propensity score matching, we conducted several analyses to assess differences between the groups. We employed chi-squared tests to compare categorical variables. The outcomes of transfusion were analyzed using a chi-squared test to determine if the patient had received a transfusion or not; this was found using ICD-10 codes, and patients who received a transfusion were placed in a group and compared between the two study populations. The same method was applied to investigate shock, endoscopy, colonoscopy, death, and AKI.

Overall, these methods provided a robust framework for evaluating the effects of DOACs on GI bleeding and related outcomes, ensuring a thorough and unbiased comparison between the pre- and post-2010 patient cohorts.

## Results

Our analysis revealed several statistically significant differences between the pre-2010 and post-2010 cohorts regarding the management and outcomes of patients with AF. Full demographic information of the included patients is outlined in Table 1 and Table 2, these were raw data numbers, and no statistical analysis was done for the numbers listed in Table 1 and Table 2. The data points listed below for the odds ratio and t-statistics were absolute values. 

**Table 1:** Demographics of patients in the post-2010 cohort

Patient demographics	
Current age, cohort 1	Mean age: 82.2
Current age, cohort 2	Mean age: 77.7
Age at index, cohort 1	Mean age: 72.6
Age at index, cohort 2	Mean age: 73.6
Race, White, cohort 1	7,156
Race, White, cohort 2	76,352
Unknown race, cohort 1	1,688
Unknown race, cohort 2	11,946
Female, cohort 1	5,091
Female, cohort 2	51,607
Unknown ethnicity, cohort 1	4,360
Unknown ethnicity, cohort 2	27,370
Not Hispanic or Latino, cohort 1	5,446
Not Hispanic or Latino, cohort 2	76,065
Male, cohort 1	5,008
Male, cohort 2	49,742

**Table 2:** Demographics of patients in the pre-2010 cohort

Patient demographics	
Current age, cohort 1	Mean age: 81.5
Current age, cohort 2	Mean age: 82.9
Age at index, cohort 1	Mean age: 73.8
Age at index, cohort 2	Mean age: 75.6
Race, White, cohort 1	5,816
Race, White, cohort 2	5,597
Unknown race, cohort 1	1,050
Unknown race, cohort 2	1,407
Female, cohort 1	4,037
Female, cohort 2	4,187
Unknown ethnicity, cohort 1	2,710
Unknown ethnicity, cohort 2	3,173
Not Hispanic or Latino, cohort 1	4,986
Not Hispanic or Latino, cohort 2	4,537
Male, cohort 1	3,901
Male, cohort 2	3,751

Table 3 displays the patient outcomes.

**Table 3:** Patient outcomes

Category	Pre-2010 cohort	Post-2010 cohort	t-statistics	95% confidence interval	P-value	Statistically significant
Total patient population	13,747	107,330	-	-	-	-
Blood transfusions	611 patients	791 patients	-0.023	(-0.031, -0.014)	<0.0001	Yes
Upper endoscopies	422 procedures	511 procedures	-0.011	(-0.018, -0.004)	0.003	Yes
Shock diagnoses	152 patients	206 patients	-0.007	(-0.011, -0.002)	0.004	Yes
Acute kidney injury	814 cases	997 cases	-0.023	(-0.033, -0.013)	<0.0001	Yes
Mortality rates	-	-	-0.003	(-0.007, 0.002)	0.234	No
Colonoscopies	-	-	-0.002	(-0.009, 0.004)	0.474	No

Blood transfusions

Patients in the post-2010 cohort required a significantly higher number of blood transfusions compared to those in the pre-2010 cohort. Specifically, 791 patients in the post-2010 group received blood transfusions, whereas 611 patients in the pre-2010 group did. The difference was significant (t-statistics=-0.023; 95% CI (-0.031, -0.014); p<0.0001). 

Upper endoscopies

The frequency of upper endoscopies was also significantly greater in the post-2010 group. There were 511 endoscopies performed in the post-2010 cohort compared to 422 in the pre-2010 cohort. This difference was statistically significant (t-statistics=-0.011; 95% CI (-0.018, -0.004); p=0.003).

Shock diagnoses

The incidence of shock diagnoses was notably higher in the post-2010 group. A total of 206 patients were diagnosed with shock in the post-2010 cohort, compared to 152 in the pre-2010 group. This difference was significant (t-statistics=-0.007; 95% CI (-0.011, -0.002); p=0.004).

AKI

AKI was more frequently diagnosed in the post-2010 cohort, with 997 cases compared to 814 in the pre-2010 group. The increase was statistically significant (t-statistics=-0.023; 95% CI (-0.033, -0.013); p<0.0001).

Mortality rates and colonoscopies

No significant differences were observed between the pre-2010 and post-2010 cohorts with respect to mortality rates during hospitalization or the need for colonoscopy. Indicating no statistically significant differences, the p-values for these comparisons were as follows: t-statistics=-0.003, 95% CI (-0.007, 0.002), and p=0.234 and t-statistics=-0.002, 95% CI (-0.009, 0.004), and p=0.474, respectively.

## Discussion

The results of this study demonstrate significant clinical implications following the introduction of DOACs in the management of AF, particularly regarding GI bleeding and other related complications. Our primary goal was to analyze data from before and after the implementation of DOACs and to assess their effects on various outcomes such as the need for transfusions, endoscopies, colonoscopies, hospital admissions, complications like shock and AKI, and mortality rates.

Our findings indicate that patients treated after the widespread adoption of DOACs post-2010 had a statistically significant increase in the need for blood transfusions compared to those managed before 2010 (791 vs. 611; p<0.0001). Blood transfusions in this context often serve as a clinical marker of the severity of bleeding, as they are typically required when patients experience significant blood loss that results in hemodynamic instability or symptomatic anemia. The increased need for transfusions indicates that bleeding events associated with DOAC use are not only more frequent but potentially more severe or difficult to control [14]. The management of bleeding in patients on DOACs can be particularly challenging due to the limited availability of reversal agents for some DOACs and the complexities involved in balancing the risks of ongoing anticoagulation with the need to control active bleeding [15]. Consequently, the increased requirement for blood transfusions observed in our study population reflects not only the prevalence of bleeding complications but also the severity and clinical burden associated with DOAC-related hemorrhages.

Similarly, the significant increase in the number of patients requiring upper endoscopies in the post-2010 group (511 vs. 422; p=0.003) indicates a proactive approach by clinicians in investigating GI complications. The heightened awareness of the bleeding risks associated with DOACs has likely prompted earlier and more frequent use of diagnostic interventions such as endoscopy. Endoscopies play a critical role in both the diagnosis and management of GI bleeding, allowing for the direct visualization of the bleeding site, assessment of the severity of bleeding, and application of therapeutic measures such as hemostatic clips, epinephrine injection, or thermal coagulation. The increase in endoscopic procedures may also reflect a shift toward more comprehensive management and preventative strategies for DOAC-associated GI bleeding, aiming to identify and address bleeding sources before they lead to more severe complications [16,17]. The Forrest classification is a widely used system to categorize the severity and risk of rebleeding in patients with upper GI hemorrhage, which is particularly relevant in the context of DOAC use. This classification system stratifies bleeding based on endoscopic findings, ranging from active spurting (Forrest I) to non-bleeding visible vessels (Forrest II) and adherent clots (Forrest III), and helps guide clinical decisions regarding the need for endoscopic hemostasis and the risk of rebleeding [18]. Research by Holster et al. [15] found that among patients on DOACs who presented with GI bleeding, a significant proportion had lesions classified as Forrest IIa or IIb, necessitating endoscopic intervention to prevent further bleeding. This classification is crucial for tailoring management strategies, as patients with higher-risk Forrest classifications may benefit from more aggressive therapeutic interventions and closer monitoring [19].

The increase in diagnoses of shock (206 vs. 152; p=0.004) further highlights that some patients are experiencing severe complications, necessitating close monitoring and prompt intervention. Immediate interventions should include timely fluid resuscitation, blood transfusions, and, if necessary, the use of reversal agents or prohemostatic treatments to counteract the anticoagulant effects [20]. The increase in shock diagnoses also emphasizes the need to develop and implement protocols that prioritize early identification and management involving the use of risk stratification tools, closer monitoring of vital signs and hemoglobin levels, and ensuring that patients and caregivers are educated on the signs and symptoms of bleeding and shock.

The rise in cases of AKI in the post-2010 group (997 vs. 814; p=0.000) also raises important considerations. Renal impairment is a known risk factor for increased bleeding in patients on DOACs, as these drugs are partially excreted through the kidneys [1]. The mechanisms by which DOACs contribute to AKI are multifaceted. One significant factor is direct nephrotoxicity; for example, dabigatran is primarily renally excreted, which can lead to its accumulation and potential nephrotoxic effects, especially in patients with impaired kidney function [21]. Additionally, DOACs increase the risk of bleeding events, including GI bleeding, which can result in hypovolemia and reduced renal perfusion, further predisposing patients to AKI [22]. Altered renal hemodynamics due to DOACs' effects on renal blood flow autoregulation may also impair glomerular filtration rate in susceptible patients [23]. The observed increase in AKI cases could therefore represent an additional complication of DOAC therapy, requiring careful dose adjustments and regular renal function monitoring.

Interestingly, the study found no significant differences between the pre- and post-2010 groups in terms of mortality rates during hospitalization or the need for colonoscopy. It is important to consider the potential role of confounding and effect-modifying factors that could influence mortality rates in the context of DOAC use. For example, improvements in the overall quality of care, including the management of comorbid conditions such as hypertension, diabetes, and heart failure, could have contributed to the stable mortality rates seen in our study, independent of the increased bleeding risk associated with DOACs [24]. Additionally, the patient population post-2010 may differ in other significant ways, such as having better access to healthcare, more frequent follow-ups, or a greater prevalence of preventative health measures, all of which could mitigate the impact of bleeding complications on mortality. Furthermore, the use of DOACs has been associated with a lower risk of intracranial hemorrhage compared to warfarin, which could potentially offset the increased risk of GI bleeding, contributing to stable overall mortality rates [25,26].

Overall, these findings underscore the need for careful monitoring and risk management strategies in patients receiving DOAC therapy, emphasizing the importance of balancing stroke prevention with the associated bleeding risks. The increased incidence of GI bleeding, shock, and AKI among patients treated after 2010 highlights the importance of individualized patient assessment and the potential need for adjusted anticoagulant dosing or selection of alternative therapies. Further research is crucial to refine clinical guidelines, improve risk stratification, and enhance patient safety in anticoagulation management. Our findings provide valuable insights into the effectiveness of DOACs in managing the risk of GI bleeding and other complications in patients with AF, underscoring the ongoing need to balance the benefits of stroke prevention against the potential harms of bleeding.

This study has several limitations that warrant consideration. First, the retrospective design inherently carries the risk of selection bias and limits the ability to establish causality. Despite using propensity score matching to minimize confounding, residual confounders may still influence the outcomes. Additionally, reliance on ICD-9 and ICD-10 codes for patient identification and outcome classification introduces the possibility of coding inaccuracies or misclassification. The exclusion of patients with certain comorbid conditions may limit the generalizability of the findings to broader patient populations. Moreover, the study did not account for variations in DOAC dosing, adherence, or the specific type of DOAC used, all of which could significantly impact bleeding risk. Lastly, the TriNetX database, while large and comprehensive, may not capture all relevant clinical nuances, such as over-the-counter medication use or detailed endoscopic findings, potentially underestimating or overestimating certain outcomes.

## Conclusions

This study highlights the complex impact of DOACs on GI bleeding in patients with AF. Our findings reveal that after the introduction of DOACs post-2010, there was a significant increase in the need for blood transfusions and upper endoscopies, along with a rise in diagnoses of shock and AKI. Specifically, patients treated after the adoption of DOACs exhibited a higher frequency and severity of GI bleeding events compared to those treated before their introduction. Despite the overall effectiveness of DOACs in reducing stroke risk, these drugs have been associated with notable increases in GI bleeding complications and related severe outcomes, but there was no significant increase in overall mortality noted. This underscores the need for ongoing vigilance in managing bleeding risks and emphasizes the importance of individualized treatment approaches to optimize patient safety. Given the observed increase in GI bleeding and related complications with DOAC use, future research should focus on several key areas. First, longitudinal studies examining the long-term outcomes of patients on DOACs could provide insights into the durability of their safety profile and the incidence of adverse events over extended periods. Additionally, research into the development of more effective and targeted reversal agents for DOACs is crucial to address bleeding emergencies more efficiently. Investigations into patient-specific factors, such as renal function and comorbid conditions, could help refine risk stratification and guide more personalized anticoagulation strategies. Furthermore, studies exploring alternative anticoagulants or dosing regimens might offer potential pathways to mitigate bleeding risks while maintaining effective stroke prevention. Ultimately, these efforts will enhance our understanding of DOACs' risk-benefit profile and contribute to improved management strategies for patients with AF.
